# Assessing the Revised Child Anxiety and Depression Scale (RCADS) in a National Sample of Danish Youth Aged 8–16 Years

**DOI:** 10.1371/journal.pone.0037339

**Published:** 2012-05-23

**Authors:** Barbara Hoff Esbjørn, Mikael Julius Sømhovd, Clara Turnstedt, Marie Louise Reinholdt-Dunne

**Affiliations:** Department of Psychology, University of Copenhagen, Copenhagen, Denmark; The University of Queensland, Australia

## Abstract

Early identification of anxiety among youth is required to prevent them from going unrecognised and untreated by mental health professionals. A precise identification of the young person’s primary difficulty is also required to guide treatment programs. Availability of a valid and easily administrable assessment tool is crucial for identifying youth suffering from anxiety disorders. The purpose of the present study was therefore to examine the psychometric properties of the Danish version of the Revised Children’s Anxiety and Depression Scale (RCADS). A total of 667 youth from community schools (4^th^ through 9^th^ grade) across Denmark participated in the study. The psychometric properties of the RCADS-_DAN_ resembled those reported in US and Europe. Within scale reliability was excellent with Chronbach’s alpha of.96. All subscales also showed good to excellent internal reliability. The study provides convincing evidence that the RCADS-_DAN_ is a valid assessment tool for screening anxiety in Danish youth.

## Introduction

### Mental health in Children and Adolescents

The ultimate goal for mental health professionals working with children and adolescents must be to increase the overall level of mental health among youths. Early identification and targeted interventions are required if this goal is to be reached. Most of the existing assessment tools have been developed in English and only a few questionnaires have been translated to Danish. So far, specific instruments for internalising disorders, that is, anxiety and depression in Danish language are scarce. Although instruments of a broader scope such as the Child Behaviour Checklist (CBCL) [1], the Strengths and Difficulties Questionnaire (SDQ) [2] and the Beck Youth Inventories have proven valid and reliable also in Danish (BYI) [3], they may not always provide the required detail in assessment, e.g., when suspecting specific anxiety diagnoses. A precise identification of the primary problem is also required to guide treatment. Unfortunately, many children suffering from anxiety are not recognised by mental health professionals and are therefore left untreated [4]. There is no reason to suspect the situation to be any different in Denmark. For instance, nearly 11% of children in the general population report emotional distress [5], however, only 5.7% of all children aged 0–18 years referred to the psychiatric system in Denmark were diagnosed with an anxiety disorder. Of these, 43% were diagnosed with a non-specified anxiety disorder [6]. A possible explanation of this situation may be the lack of accessible screening tools for anxiety and depression that also delineates the specific anxiety diagnosis and depression. Availability of a valid and easily administrable assessment tool is crucial for improving the validity of a given diagnosis [7], [8]. As lack of identification and treatment of anxiety in childhood increases the risk of psychiatric disturbances in adulthood [9], and as anxiety disorders tend to be stable throughout life [10], this is an area that must be taken seriously if we are to improve mental health among our youth. Gender differences have been reported, with girls having twice as big a risk as boys for experiencing anxiety disorders [11], so these factors must be taken into consideration in the process of identifying anxiety in youth.

### Identification of Anxiety Disorders

Questionnaires building on the DSM-IV criteria have been developed. A very widely used questionnaire is the Spence Childreńs Anxiety Scale (SCAS) [12], measuring five different anxiety disorders. Based on a revision of the SCAS, the Revised Children’s Anxiety and Depression Scale (RCADS) [13] was created. It is designed to assess clinical syndromes of anxiety as well as depression. The RCADS provides two total scores, and 6 subscales: Separation Anxiety Disorder (SAD), Social Phobia (SoP), Obsessive Compulsive Disorder (OCD), Panic Disorder (PD), Generalized Anxiety Disorder (GAD) and Major Depressive Disorder (MDD). One total score is a sum of all anxiety subscales, which gives an overall index of anxiety levels. The other is a sum of all subscales, and it provides an estimate of the total level of internalising symptoms. The internal consistency of the RCADS subscales is high, with Cronbach alphas ranging from 0.78 (SAD) to 0.88 (GAD) [13]. Correlations between RCADS and the Revised Childreńs Manifest Anxiety Scale subscales (RCMAS) [14] range from 0.49 to 0.68 [15], and correlations with SCAS range from 0.50 to 0.61 [16]. Similar results are found regarding the correlation between the RCADS depression subscale and the Childreńs Depression Inventory (CDI) [17], where the correlation was reported to be 0.80 [18].

Furthermore, analyses of the factor structure of the RCADS show that a 6-factor model consistent with mentioned anxiety disorders and depression provided the best fitting model [13], [15]. The 6-factor model was also reproduced in a confirmatory factor analysis in a large community sample of 10–12 year old children (the TRAILS study, *N* = 2230) [10]. However, not all studies report findings consistent with this structure. In contrast, Muris, Meesters and Schouten [19], found that the 47-item RCADS did not load convincingly on the hypothesised 6-factor structure. Rather, a reduction to 25 items, loading on a 5-factor structure, provided a better model fit for the data. The removed items provided information on the OCD subscale. The psychometric properties of the RCADS-25 were comparable to those obtained on the original version of the RCADS with satisfactory internal consistency (Cronbachs alphas between 0.65 and 0.83). Despite these inconsistencies, the above mentioned studies all reported that RCADS differentiated between subtypes of anxiety and depression. When applying a latent class analysis to identify homogenous subgroups in a large community sample, the RCADS, however; failed to distinguish between any of the different types of anxiety [20]. Nonetheless, evidence is building that the RCADS provides valid and reliable information on anxiety and depression in children and adolescents.

### Impact of Gender and Age on Levels of Anxiety and Depression

Studies assessing the impact of gender and age on anxiety and depression have consistently shown that girls report higher levels of anxiety and depression than boys [11], [21]. Whereas the increased incidence of anxiety disorders among girls is reported irrespectively of age, the preponderance of depression in girls is found from early adolescence and onwards [21]. Overall, the prevalence of depression increases from childhood to adolescence, whereas, some anxiety disorders (e.g., separation anxiety) decrease with age and others increase (e.g., generalised anxiety disorder) [8].

These overall findings have been reproduced in studies applying the RCADS. Analyses of gender differences have consistently shown that girls display higher scores than boys on all RCADS subscales, indicating higher levels of anxiety and depression [10], [13], [15], [18]–[19]. Analyses of age, however, are less clear. Although two studies have reported significant reductions in symptom level with increasing age [10], [19], van Oort et al found that most anxiety symptoms tended to increase again from middle adolescents after the initial decrease in symptom level [10].

### Purpose of the Present Study

Questionnaires targeting the specific anxiety diagnoses have been developed internationally, however, only few of these have been translated and assessed on a Scandinavian sample. The purpose of the present study was therefore to examine the psychometric properties of the Danish version of the RCADS. The validity and factor structure were assessed on a national sample of youth (*N* = 667) aged 9 to 17 years. Gender specific normative data are provided to ensure the availability of a valid and reliable childhood anxiety assessment tool for clinicians in Denmark.

## Methods

### Participants

The participants in the study comprise a national sample and were recruited from community schools (4^th^ through 9^th^ grade) in Denmark. A missing data pattern analysis revealed no systematically missing patterns. Varying across the RCADS items where between 6 and 20 user missing values and thus we decided to replace these by the linear trend of that particular data-point. A total of 667 participating youth answered the RCADS questionnaire, but 49 did not report their gender. Of those reporting gender (*n = *618), 333 were girls (53.9%) and 285 (46.1%) were boys. Approximately 10% of the sample did not report their age. As children not reporting age were not expected to score differently from the remaining children, all cases were kept in the analyses. The only exception was analyses of age and gender (*n* = 422). The sample had a mean age of 12 years and 6 months (SD = 1 year, 8 months). Further, there were no statistical differences regarding parental education, parent status or family income between included and excluded children.

### Procedure

An information letter was sent to *all* schools in Denmark with more than 3 classes at each grade. A school was considered as enrolled when the headmasters and school-boards gave their consent. Subsequently, information letters where delivered to the families via the teachers in each class, who also collected the individual informed consent forms. A total of 210 schools were contacted and 19 chose to participate. Data on participating families was compared to that of the overall population obtained through the central governmental agency for statistics – Statistics Denmark. In our sample, 72% of the children were living with both biological parents compared to 75% in the overall population. A larger percentage of the mothers in our sample had a medium length education than the overall population of mothers, with 9% vs. 33% having no or short education, 68% vs. 37% having a medium length education and 20% vs. 29% having a long education. As the participating schools were evenly scattered across Denmark with both urban and rural areas represented, and the educational level of the mothers did not indicate markedly higher percentages of longer educational levels, the sample may be considered to be relatively representative of the Danish population of children. Norms based on the sample would in case of bias most likely underestimate the true number of difficulties as found in most standardization studies. A self-report test battery was administered to the children when in school. To ensure anonymity for the child and thereby reduce the likelihood of peer-pressure influencing the answers provided, the children were placed at single tables in a large room with distance between all tables. Of relevance for the present study were RCADS and SCARED-R. Project staff was present during the testing session and helped the youth as needed. By the end of the testing, all youth received a small gift as appreciation for their efforts.

### Ethical Statement

The study has been subjected to ethical review at the Institutional Review Board at the University of Copenhagen, and complies with current ethical standards in assessment and treatment in Denmark for children enrolled research studies. Written informed consent to participate was obtained from all parents of participating youth.

### Measures

#### Revised Children’s Anxiety and Depression Scale (RCADS)

RCADS [15] consists of 47 items developed to measure DSM-IV relevant symptoms of anxiety disorders (GAD, SAD, SoP, Panic disorder, OCD) and Depression in children. It is scored on a 4-point scale (0 = never, 1 = sometimes, 2 = often and 3 = always). The Danish RCADS was translated and back-translated as part of previous research [22]. This study is a part of its validation process, and thus the Screen for Child Anxiety Related Emotional Disorders (SCARED-R) was examined for convergent validity purposes.

#### Screen for Child Anxiety Related Emotional Disorders (SCARED-R)

The SCARED-R [23], [24] consists of 69 items scored on a 3-point scale (0 = almost never, 1 = sometimes, and 2 = often). It has been reported to have satisfactory test-retest reliability and good internal consistency [24]–[26]. Furthermore, it is sensitive to effects of treatment and discriminates well between anxiety disorders and other problems, e.g., major depressive disorder, attention-deficit and hyperactivity disorder, and oppositional-defiant disorder [25], [26].

### Data Analysis

Reliability assessment was employed. Also, to test construct validity, discriminant and convergent validity of the RCADS, subscales were compared with SCARED-R data using Pearson correlations. Furthermore, t-tests for independent means and Pearson correlations were used to assess age and gender differences. Finally, a confirmatory factor analysis was carried out based on the a priori factor structure from the Weiss and Chorpita [27] user manual for RCADS (see appendix at http://ccap.psy.ku.dk/testmateriale/for further detail). As it has been reported that ordinal factor indicators potentially lead to spurious chi square values we chose to disregard the chi statistic as recommended by Jöreskog and Sörbom [28]. Adherently, the model fit was evaluated using three fit indices: (a) goodness-of-fit index above.95, (b) adjusted goodness-of-fit index above.95, and (c) root mean square residual below 0.05 to suggest a good fit to data [28].

## Results

### Internal Consistency and Validity

Overall, within scale reliability was excellent with a Chronbach’s alpha of.96. None of the items influenced the alpha noticeable in a deletion of items procedure (<.001). All subscales also showed good to excellent internal reliability (GAD:.90, MDD:.86, SA:.75, SOC:.75, OCD:.77, PA:.84.). Pearson correlations were used to test the convergent validity of all RCADS anxiety subscales with the SCARED subscales. This resulted in moderate to strong correlations between equivalent subscales (see table 1).

**Table 1:** Correlations between RCADS and SCARED-R anxiety subscales.

		RCADS			
	SAD	GAD	SoP	PD	OCD
**SCARED-R**					
SAD	.56				
GAD		.53			
SoP			.43		
PD				.58	
OCD					.56

Pearson Correlation, all *p*<.001; N = 667.

SAD = Separation anxiety disorder; GAD = Generalised anxiety disorder; SoP = Social Phobia, PD = Panic disorder, OCD = Obsessive compulsive disorder.

### Gender and Age Differences

To measure gender differences, independent samples *t*-tests were performed on RCADS total score and on all the subscales. There was a significant difference in the total internalizing scores for girls (Mean = 30.3±18.8) and boys (Mean = 22.3±16.3). Girls also reported higher levels of anxiety and depression than boys on all subscales (see table 2). The mean gender difference for the total internalizing scale was 7.9 with a medium effect size, partial *η^2^* = .07. The effect remained significant on all subscales. However, Pearson correlations revealed no significant association between RCADS and age, *p* = .19.

**Table 2:** Gender differences on RCADS total scores and subscale scores.

RCADS	Mean (SD)		*T*	95% CI	
	Male(*n* = 285)	Female(*n* = 333)		Lower	Upper
Total internalising	22.3 (16.3)	30.3 (18.8)**	5.61	5.20	10.80
Total anxiety	17.5 (13.3)	24.0 (14.8)**	5.73	4.28	8.77
Generalised anxietydisorder	3.3 (2.7)	4.6 (3.2)**	5.45	.84	1.78
Social phobia	6.2 (4.4)	8.5 (4.8)**	6.26	1.60	3.07
Separation anxiety	2.0 (2.3)	3.0 (2.9)**	5.00	.64	1.46
Obsessive compulsivedisorder	2.8 (2.7)	3.3 (2.7)*	2.55	.13	.97
Panic disorder	3.2 (3.4)	4.5 (4.0)**	4.24	.69	1.88
Depression	4.8 (3.6)	6.2 (4.7)**	4.45	.82	2.13

Note: ** p<.001; * p<.05; CI = Confidence Interval.

### Confirmatory Factor Analysis

The a priori RCADS six factor model demonstrated an adequate fit to the data with RMSEA = .05; CFI = .95; and TLI = .94). The a priori model is hence confirmed as a 6-factor structure equivalent to the RCADS’s six subscales in the Danish translation.

### Percentile Distribution of RCADS-_DAN_ Scores

Percentiles were calculated for a total internalizing score and a total anxiety score (see table 3). As there was a significant effect of gender, with girls scoring higher than boys on all subscales, we present the scores according to gender as well as to the overall sample. Furthermore, gender specific percentiles were calculated for each subscale. Data is presented in Appendix 1 which is posted on our web-site http://ccap.psy.ku.dk/testmateriale/along with scoring guides and the Danish version of RCADS.

**Table 3:** Percentile scores for Total internalizing and Total anxiety on the RCADS-_DAN_.

*Percentile*	Total group*N = 618		GirlsN = 333		BoysN = 285	
	TotInt	TotAnx	TotInt	TotAnx	TotInt	TotAnx
10	6	4	10	7	4	3
20	11	9	15	13	8	6
30	16	13	23	17	12	9
40	23	18	27	21	16	12
50	26	21	27	21	20	15
60	27	21	28	23	26	21
70	30	24	35	27	27	21
80	38	30	42	34	34	26
90	49	38	54	45	45	36
95	59	49	68	53	54	43
97.5	74	55	81	62	60	50
99	92	73	97	78	71	57

Note: * with gender reported; Tot Int = Total scale internalizing; Tot Anx = Total scale anxiety.

## Discussion

The aim of this study was to examine the psychometric properties of the Danish version of the RCADS. The internal consistency of the RCADS-_Dan_ was excellent. This is in accordance with previous studies of the RCADS in other languages [13]. The convergent validity of the RCADS-_Dan_ anxiety subscales compared to the SCARED-R subscales showed moderate to good associations. This finding is also corroborated by previous studies of both the original RCADS with 47 items [15] and the shorter 25 item version [19]. We were, however, not able to examine the convergent validity of the depression subscale of the RCADS-_Dan_ due to lack of a separate measure of depression in the study design. Further analyses should be conducted to test this subscale, however, previous studies have all reported a satisfactory convergent validity for the depression scale along with that of the anxiety subscales [10], [13], [15].

Analyses of the factor structure of the RCADS-_Dan_ confirmed six different and distinct factors consistent with the 6 distinct diagnostic categories; Separation Anxiety Disorder, Social Phobia, Obsessive Compulsive Disorder, Panic Disorder, Generalized Anxiety Disorder and Major Depressive Disorder, as suggested by Chorpita and colleagues [15]. Although our results are not supported by those of Ferdinand et al. [20], it remains a fairly robust finding that the RCADS provides either 5 or 6 factors [13], [19].

### Gender Differences on RCADS

Our findings regarding gender differences are supported by previous reports [10], [19]. Overall, girls scored higher on anxiety and depression than boys. Previous studies of normative samples from the US, Australia and the Netherlands have reported separate mean subscale scores for girls and boys. When calculating a total mean internalizing score based on the 47 item RCADS for these samples, the US mean for girls presented the highest levels of internalizing disorders (Mean: US = 43.3 [18]; Australian = 34.8 [18]; Netherlands = 26.3 [19]; Denmark = 30.3). The means from the three other samples are more in line with each other. Also, boys in the US presented the highest scores (Mean: US = 37.5; Australian = 32.0; Netherlands = 20.3; Denmark = 22.3). The mean score of the Danish boys resembles that of boys from the Netherlands. This finding is in accordance with previous studies reporting higher overall mean scores in US samples compared to Danish samples [22]. However, Danish samples norms on similar tests are reported to be in accordance with Dutch samples [29]. This finding also contributes to the conclusion that the Danish version of the RCADS full-fills psychometric criteria for a valid and reliable assessment tool.

### The Applicability of the RCADS-_DAN_ as a Screening Instrument

Our aim was to ensure the availability of a cost-effective and valid Danish assessment tool for children with anxiety and depression. The sample selected to test the RCADS-_DAN_ was evenly distributed across the country and both urban and rural schools were enrolled in the study. However, one cannot rule out that children who participated may come from socially more advantaged homes than those who did not participate. If the participating sample is more advantaged overall than the general population, norms may only provide minimum figures regarding difficulties. However, this only strengthens our finding that an application of the reported percentiles in the present study will report true findings of emotional difficulties in youth.

As the data are derived from a normative sample using only a self-report questionnaire one cannot calculate clinical cut-off scores with accurate precision at present. Further studies investigating the distribution of scores in a clinical sample are required. However, the present study does provide valuable information about the RCADS as a screening instrument, which may be applied for screening of children and adolescents in Denmark for anxiety and depression. Due to the gender differences the percentiles are reported separately for girls and boys on a total internalising score and on each of the subscales (see appendix at http://ccap.psy.ku.dk/testmateriale/). The reported gender specific percentiles enhance the applicability of the RCADS-_DAN._ This will make administration of the test easier for professionals, increasing the likelihood of a correct identification of youth, who are in need of further assessment and treatment. As precise cut-off scores are yet to be created, we encourage professionals using the RCADS-_DAN_ to engage in further assessment of the youth scoring above the 70^th^ percentile. Further assessment should also provide a detailed description of the specificity of the anxiety disorder symptoms, which will provide important information for the creation of an individual treatment plan.

Despite above mentioned limitations, the present study adds to the broadening of the psychometric support of the RCADS to a wider population of nonclinical school children. It also provides useful normative data which allow for effective initial detection of youth with anxiety and depression in school psychologist and general practitioner settings. Continued research on larger clinical populations is needed and would provide beneficial information about the usability of RCADS-_DAN_ in psychiatric in and outpatient facilities.

### Conclusion

The present study provides convincing evidence that the RCADS-_DAN_ is a valid assessment tool for screening of anxiety and depression in Danish youth. In a large national sample (N = 667) the psychometric properties of the RCADS-_DAN_ were found to resemble those reported in previous studies.
